# Volumetric and Diffusion Abnormalities in Subcortical Nuclei of Older Adults With Cognitive Frailty

**DOI:** 10.3389/fnagi.2020.00202

**Published:** 2020-07-28

**Authors:** Mingyue Wan, Rui Xia, Huiying Lin, Pingting Qiu, Jianquan He, Yu Ye, Jing Tao, Lidian Chen, Guohua Zheng

**Affiliations:** ^1^College of Rehabilitation Medicine, Fujian University of Traditional Chinese Medicine, Fuzhou, China; ^2^Fujian Key Laboratory of Rehabilitation Technology, Fujian University of Traditional Chinese Medicine, Fuzhou, China; ^3^College of Nursing and Health Management, Shanghai University of Medicine and Health Sciences, Shanghai, China

**Keywords:** cognitive frailty, subcortical nuclei, diffusion tensor imaging, volume, correlation

## Abstract

**Background**: Cognitive frailty (CF) is defined as the simultaneous presence of physical frailty and cognitive impairment among older adults without dementia. Previous studies have revealed that neuropathological changes may contribute to the degeneration of subcortical nuclei in the process of cognitive impairment. However, it is unclear in CF. The aim of this study is to investigate the changes in subcortical nuclei in older adults with CF and their relationship with cognitive decline and physical frailty.

**Methods**: A total of 26 older adults with CF and 26 matched healthy subjects were enrolled. Cognitive function and physical frailty were assessed with the Montreal Cognitive Assessment (MoCA) scale (Fuzhou version) and the Chinese version of the Edmonton Frailty Scale (EFS). Volumetric and diffusion tensor imaging (DTI) parameters of subcortical nuclei were measured with structural and DTI brain magnetic resonance imaging (MRI) and compared between groups. Partial correlation analysis was conducted between subcortical nuclei volumes, MoCA scores, and physical frailty indexes.

**Results**: Significant volume reductions were found in five subcortical nuclei, including the bilateral thalami, left caudate, right pallidum, and accumbens area, in older adults with CF (*P* < 0.05), and the bilateral thalami was most obvious. Decreased fractional anisotropy and relative anisotropy values were observed only in the left thalamus in the CF group (*P* < 0.05). No group differences were found in apparent diffusion coefficient (ADC) values. The MoCA scores were positively correlated with the volumes of the bilateral thalami, right pallidum, and accumbens area (*P* < 0.05). Negative correlations were found between the physical frailty index and the volumes of the bilateral thalami, caudate, pallidum, and right accumbens area (*P* < 0.05).

**Conclusion**: Microstructural changes occur in the subcortical nuclei of older adults with CF, and these changes are correlated with cognitive decline and physical frailty. Therefore, microstructural atrophy of the subcortical nuclei may be involved in the pathological progression of CF.

## Introduction

Cognitive frailty (CF) was first defined in 2013 by the International Academy on Nutrition and Aging (I.A.N.A.) and the International Association of Gerontology and Geriatrics (I.A.G.G.; Kelaiditi et al., 2013). Cognitive frailty has a heterogeneous clinical manifestation characterized by the simultaneous presence of both physical frailty and cognitive impairment among older adults without dementia. Epidemiological surveys based on community people estimated the prevalence rate of CF to be 1.0% to 22.0% (Shimada et al., 2016; Feng et al., 2017), whereas in the clinical setting, the figure was much higher at 10.7% to 40% (Malek et al., 2019). Cognitive frailty has been identified to be associated with an increased risk of severe adverse events in older people, such as dementia and disability and even mortality. Therefore, CF is recognized as an important threat to healthy aging. With the growing understanding of CF as an important clinical syndrome, identifying biomarkers of pathophysiological changes has become paramount. Furthermore, it is helpful to map its biological markers for the development of secondary preventive treatments for this disorder.

Clinically, the judgment of CF is typically established using tests of physical and mental status, including the Clinical Dementia Rating (CDR) and Montreal Cognitive Assessment (MoCA) or Mini-Mental State Examination (MMSE; Won et al., 2018). With the development of morphometric magnetic resonance imaging (MRI) technology, robust imaging biomarkers for some diseases related to brain damage have educed the potential for clinical impact. For example, MRI studies based on voxel-based morphometry analysis have established the association of cognitive impairment and morphometric changes in the brain. Previous studies have reported reductions in the volumes of the whole hippocampus, medial temporal lobe, and entorhinal cortex in the brains of older adults with cognitive impairment compared to healthy subjects (Anatürk et al., 2018; Gu and Zhang, 2019). Cortical gray matter atrophy first occurs in the temporal cortex and is followed by occipital, parietal, and frontal atrophy along a temporospatial gradient with the progression of cognitive decline (Fennema-Notestine et al., 2009). Considering that microstructural changes likely precede macrostructural changes, studies have revealed that the degeneration of subcortical nuclei also contributes to neuropathological changes in the process of cognitive impairment (Tentolouris-Piperas et al., 2017). However, compared to macroscopic measurements, the underlying microstructural changes seem complicated and heterogeneous (Gong et al., 2017).

Diffusion tensor imaging (DTI) can be used to quantify microscopic white/gray matter integrity not detectable on conventional MRI and is sensitive for detecting microstructural volumetric impairment through the intrinsic properties of water diffusion (Mori and Zhang, 2006). DTI has helped to explain neuropathological mechanisms for different neuropsychiatric conditions (Cherubini et al., 2010). Several studies have used DTI to explore the microstructural changes in subcortical nuclei in conditions associated with cognitive decline (Eustache et al., 2016; Mak et al., 2017). Moreover, few studies have focused on CF. However, there have been reports of different patterns of degeneration that were compatible with either mild cognitive impairment or physical frailty. The aim of the present study was to investigate microstructural volumetric changes in brain DTI indexes in older adults with CF and to compare their trajectories with those of healthy older adults.

## Materials and Methods

### Participants

Twenty-six older adults with CF and 26 matched healthy controls (HCs) participated in this study. All participants were right-handed native Chinese speakers. The CF participants were all recruited from the communities in Fuzhou City, Fujian Province, China, and the HC participants were recruited from the same communities. Written informed consent was obtained from all participants before data collection. This study was approved by the ethics committee of the Second People’s Hospital affiliated with Fujian University of Traditional Chinese Medicine. All CF participants met the following inclusion criteria: (1) Chinese version of Edmonton Frailty Scale (EFS) ≥5 points; (2) Fuzhou version MoCA score ≤26 points; (3) age ≥60 years old; and (4) CDR score = 0.5 (i.e., did not meet the criteria for dementia). The controls did not have CF syndrome, which was confirmed using the assessment of EFS and MoCA scale. Individuals who met the following conditions, which were assessed based on medical records, were excluded: (1) had hypertension and uncontrollable blood pressure (systolic blood pressure >160 mm Hg or diastolic blood pressure >100 mm Hg after taking drug); (2) had a history of alcohol and drug abuse; (3) had a history of mental illness (such as personality disorder, schizophrenia, etc.), severe depression (Baker Depression Scale >10 points), severe aphasia and audiovisual impairment, severe organ failure, cerebral hemorrhage, cerebral infarction, history of myocardial infarction, history of coronary heart disease, musculoskeletal disease, and other sports contraindications; (4) had metal implants (such as fixed metal dentures, pacemakers, etc.); and (5) were not suitable for MRI scans.

### MRI Data Acquisition

The structural MRI and diffusion tensor MRI data for all participants were collected at Fujian Province Rehabilitation Hospital. MRI scans were acquired with a Siemens Prisma 3.0-T magnetic resonance scanner (Siemens Medical System, Erlangen, Germany) and a Siemens 64-channel head-neck joint coil. The subject lay back on the scanning bed, his/her head was properly fixed, and rubber earplugs were used to reduce the noise impact of the machine. High-resolution structural images were acquired by using a three-dimensional T1-weighed magnetization-prepared rapid acquisition of a gradient echo sequence with the following parameters: repetition time (TR) = 2,300 ms, echo time (TE) = 2.27 ms, flip angle = 8°, slice thickness = 1.0 mm, field of view (FOV) = 250 × 250 mm, matrix = 256 × 256, voxel size = 0.98 × 0.98 × 1 mm^3^, and number of slices = 160. For DTI, the following parameters were used: TR = 8,000 ms, TE = 64 ms, FOV = 224 × 224 mm, slice thickness = 2.0 mm, gap = 0 mm, slice number = 75, and slice order = interleaved.

### Image Processing

The original data from the magnetic resonance image machine were transformed from DICOM format to nifti format file and classified into T1 images and DTI images by MRIconvert software (version 2.0 Rev. 235^1^). T1 image analysis was carried out with FreeSurfer software (version 6.0.0^2^). DTI analysis was carried out with FSL software (version 5.0.10^3^).

### Volumes of the Subcortical Nuclei Analysis

The T1 image preprocessing procedure included image format conversion, image quality check, Talairach transformation (this computes the affine transform from the original volume to the MNI305 atlas), intensity normalization, skull stripping, segmentation of gray and white matter, tessellation of the gray and white matter boundary, automatic subcortical segmentation, automatic topology fixer, and smoothing.

For comparisons of the volumes of the subcortical structures between the groups, the automatic extraction technology of FreeSurfer was used. FreeSurfer segments the brain into several regions that have a unique id. Through the segmentation id, we could identify the nuclei of interest and extract their information. The volumes of the subcortical nuclei, including the thalamus, caudate, putamen, pallidum, hippocampus, amygdala, and accumbens area of both sides, were extracted.

### Diffusion Parameters of the Subcortical Nuclei

The DTI image data preprocessing procedure included image format conversion, image quality check, eddy current and motion correction, skull stripping, DTI tensor [including fractional anisotropy (FA), apparent diffusion coefficient (ADC), relative anisotropy (RA)] construction, registration of low b images to the same-subject anatomical used affine registration and deformed registration, and map FA to Talairach space to obtain DTI parameters of the subcortical nucleus in the same space with volume extraction. Then, the same subcortical nuclei segmentation id was used as the last step to extract the FA, ADC, and RA values of subcortical nuclei with volume differences between the two groups.

### Statistical Analysis

Statistical analysis was performed using the Statistical Package for Social Sciences for Windows version 21.0 (IBM Corp., Armonk, NY, USA), and *P* < 0.05 was considered significant. Continuous variables were expressed using the mean and standard deviation for the normal distribution or median and its interquartile range for a nonnormal distribution and were analyzed using Student *t*-test or Mann–Whitney *U* test, respectively. Categorical variables were described as frequencies and were analyzed using the *χ*^2^ test.

Volumetric and DTI parameters of subcortical nuclei in the two groups were compared by independent-sample *t*-tests. To identify brain structural changes related to the frailty index and MoCA, we assessed nuclei volume differences between groups using partial correlation analysis. Age, sex, education years, and Beck Depression Inventory scores were also entered as covariates.

## Results

### The Baseline Characteristics of the CF Participants and Healthy Controls

The average MoCA score in the CF group was significantly lower than that in the healthy control group. The frailty index in the CF group was significantly higher than that in the healthy control group. There were no significant differences between the two groups in gender, age, years of education, or Beck Depression Inventory scores (*P* < 0.05; Table 1).

**Table 1:** Demographic and cognitive and physical frailty data of two groups.

	Cognitive frailty	Health control	*t*	*P*
Number	26	26		
Sex (male/female, *n*)*	13/13	13/13		1.000
Age (years)^#^	65.42 ± 5.147	65.38 ± 4.7	0.028	0.978
Edu (years)^#^	9.77 ± 3.983	10.96 ± 3.243	−1.184	0.242
BDI (scores)^#^	3.96 ± 1.928	4.62 ± 1.134	−1.49	0.144
MoCA (scores)^#^	19.31 ± 3.056	26.77 ± 0.863	−11.98	<0.001
FI (scores)^#^	5.54 ± 0.761	2.19 ± 1.266	8.263	<0.001

Edu, education year; BDI, Beck Depression Inventory; MoCA, Montreal Cognitive Assessment; FI, Frailty index. *Chi-square test was used for data analysis. ^#^t-test was used for data analysis. P-values for cognitive frailty vs. health control group comparison.

### Subcortical Nuclei Volume

**Figure 1:**
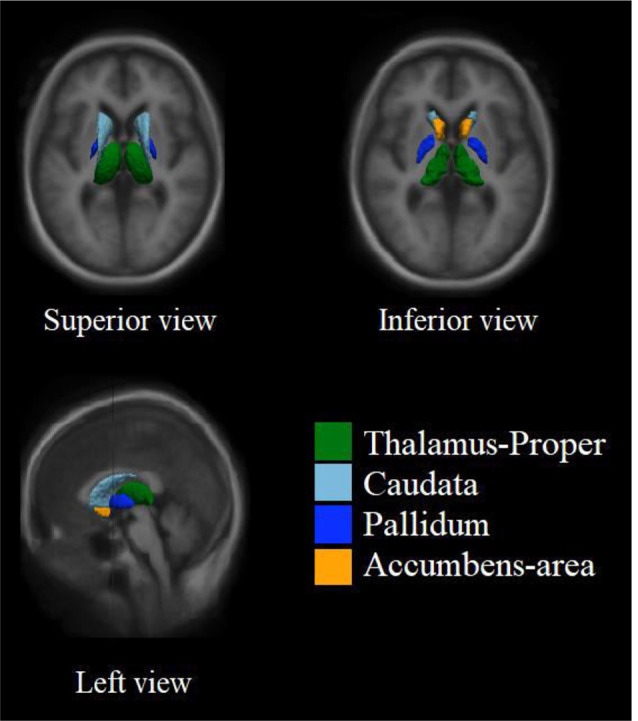
Example location of four subcortical nucleus in the brain. The spatial distribution of subcortical nucleus with volume differences in the standard brain was showed. The thalamus, located on both sides of the third ventricle, is the largest of these subcortical nucleus. Pallidum is located in the anterior inferior part of thalamus. The caudate is located in the front and upper part of pallidum. The accumbens is located in the lower part of caudate and the anterior part of pallidum.

**Figure 2:**
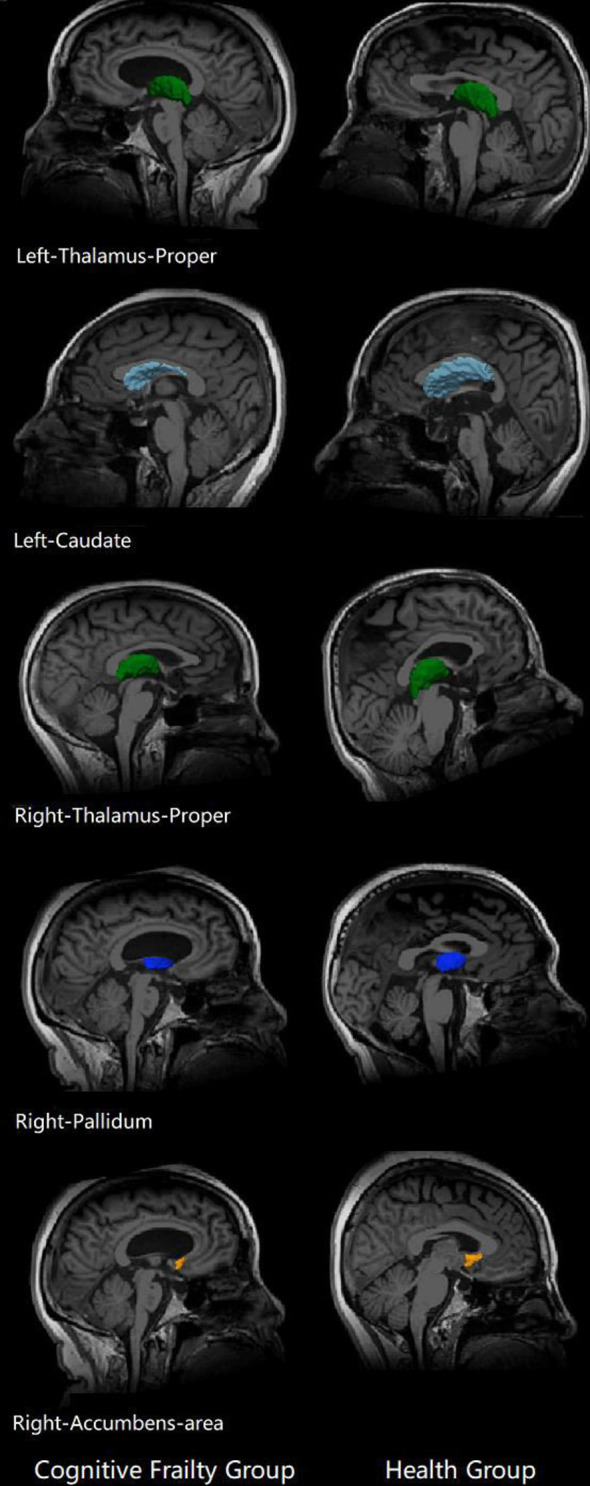
Subcortical nuclei with differences in the two groups. Brain structural changes associated cognitive frailty. Cognitive frailty group showed a decrease in the volume of subcortical nucleus compared with health group.

Compared to the healthy control group, the CF group showed decreased volume in five brain nuclei, including the left thalamus, left caudate, right thalamus, right pallidum, and right accumbens area (*P* < 0.05), and there was the most obvious volume difference in the thalamus (Figures 1, 2, Table 2).

**Table 2:** Volume of four subcortical nucleus between two groups.

	CF (volume)	HC (volume)	MD	95% CI of MD	*P*
Left-Thalamus	6,312.08 ± 527.71	6,719.65 ± 606.56	−407.57	−724.27, −90.87	0.013
Left-Caudate	3,086.25 ± 297.31	3,296.13 ± 357.49	−209.88	−393.04, −26.73	0.026
Left-Putamen	4,488.97 ± 550.071	4,594.08 ± 458.65	−105.11	−387.23, 177.01	0.458
Left-Pallidum	1,842.26 ± 212.50	1,947.05 ± 206.64	−104.79	−221.55, 11.96	0.077
Left-Hippocampus	3,661.82 ± 350.073	3,702.47 ± 358.47	−40.64	−238.01, 156.73	0.681
Left-Amygdala	1,546.19 ± 160.35	1,549.1 ± 230.30	−2.91	−113.45, 107.63	0.958
Left-Accumbens	421.34 ± 75.00	428.90 ± 62.18	−7.56	−45.94, 30.82	0.694
Right-Thalamus	6,068.14 ± 706.36	6,487.33 ± 501.10	−419.19	−760.34, −78.05	0.017
Right-Caudate	3,110.63 ± 334.18	3,290.38 ± 366.69	−179.75	−375.18, 15.67	0.071
Right-Putamen	4,382.33 ± 696.84	4,505.57 ± 523.34	−123.24	−466.52, 220.04	0.474
Right-Pallidum	1,796.72 ± 244.54	1,970.20 ± 197.54	−173.48	−297.31, −49.65	0.007
Right-Hippocampus	3871.41 ± 386.159	3872.01 ± 410.20	−0.60	−222.51, 221.32	0.996
Right-Amygdala	1,721.5 ± 210.701	1,680.52 ± 226.51	40.98	−80.88, 162.84	0.502
Right-Accumbens	451.97 ± 53.86	482.32 ± 45.68	−30.35	−58.17, −2.54	0.033

*MD, Mean difference; CI, confidence interval of difference. Value of volume was presented by mean ± standard deviation, the unit of volume is cubic millimeter*.

### DTI Parameters

**Figure 3:**
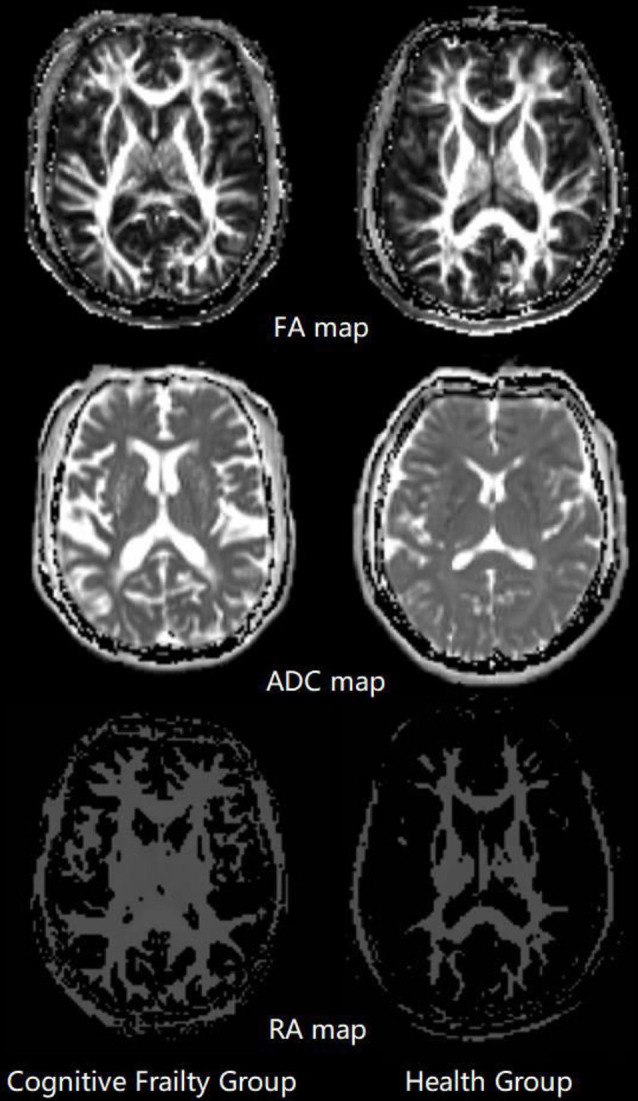
Fractional anisotropy (FA), apparent diffusion coefficient (ADC), relative anisotropy (RA) map comparison between two groups. The mean values of FA, ADC and RA were computed within thalamus, caudate nucleus, globus pallidus and nucleus accumbens for individual subjects.

Decreased FA and RA values were observed in the left thalamus in the CF group compared to the healthy control group (*P* < 0.05). No group significance was found in the ADC values of any subcortical nuclei (*P* > 0.05; Figure 3, Table 3).

**Table 3:** FA, ADC and RA of four interested subcortical nucleus between two groups.

	Group	FA	*P*	ADC (*1,000)	*P*	RA (*1,000)	*P*
Left-Thalamus	CF	0.277 ± 0.015	0.03	0.923 ± 0.043	0.792	6.9 ± 0.334	0.03
	HC	0.291 ± 0.030	0.927 ± 0.060	7.242 ± 0.706
Left-Caudate	CF	0.170 ± 0.016	0.248	0.981 ± 0.063	0.239	4.269 ± 0.403	0.333
	HC	0.180 ± 0.043	0.958 ± 0.076	4.485 ± 1.050
Left-Pallidum	CF	0.357 ± 0.031	0.136	0.738 ± 0.050	0.583	8.6 ± 0.812	0.129
	HC	0.337 ± 0.057	0.746 ± 0.051	8.096 ± 1.452
Left-Accumbens	CF	0.181 ± 0.017	0.937	0.842 ± 0.070	0.829	4.277 ± 0.378	0.875
	HC	0.180 ± 0.030	0.838 ± 0.057	4.25 ± 0.785
Right-Thalamus	CF	0.272 ± 0.017	0.219	0.942 ± 0.058	0.629	6.808 ± 0.375	0.28
	HC	0.279 ± 0.023	0.935 ± 0.056	6.942 ± 0.505
Right-Caudate	CF	0.170 ± 0.023	0.528	0.981 ± 0.106	0.7	4.273 ± 0.616	0.782
	HC	0.174 ± 0.026	0.969 ± 0.109	4.319 ± 0.582
Right-Pallidum	CF	0.329 ± 0.033	0.777	0.762 ± 0.057	0.61	7.854 ± 0.784	0.779
	HC	0.333 ± 0.062	0.754 ± 0.051	7.95 ± 1.550
Right-Accumbens	CF	0.182 ± 0.017	0.396	0.827 ± 0.060	0.211	4.277 ± 0.377	0.476
	HC	0.188 ± 0.028	0.808 ± 0.049	4.388 ± 0.697

*Group CF, cognitive frailty group; group HC, healthy group; FA, fractional anisotropy; ADC, apparent diffusion coefficient; RA, relative anisotropy. Value of FA, ADC and RA was presented by mean ± standard deviation*.

### Correlation Between Subcortical Nuclei Volumes, MoCA Scores, and Frailty Index Scores

**Figure 4:**
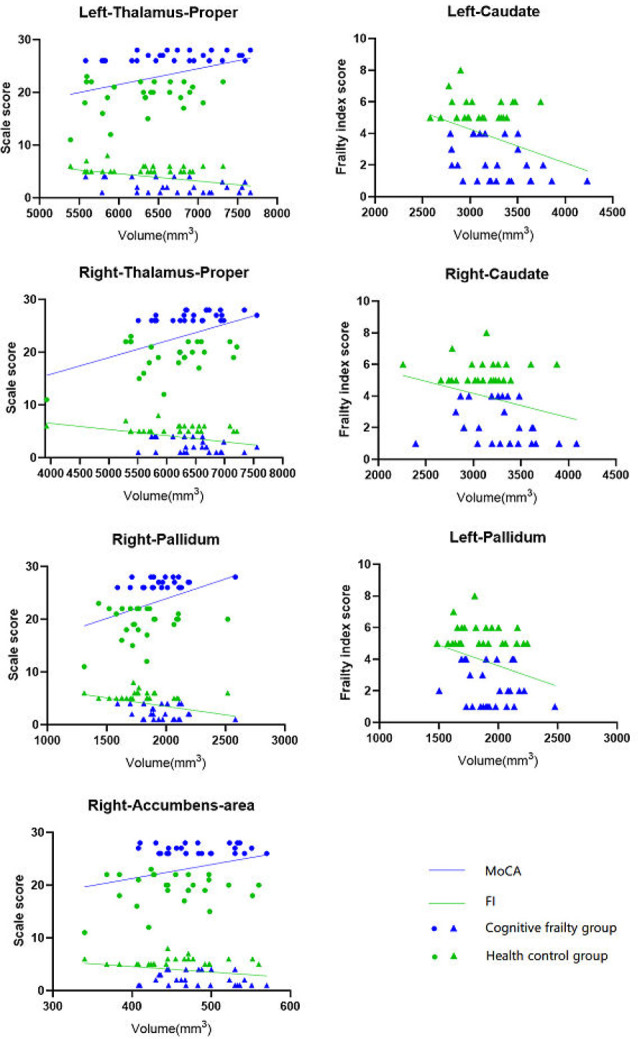
Correlation between subcortical nucleus volume and MoCA, FI values after adjusted for age, sex and education. The nucleus volume in left and right thalamus-proper, right pallidum and right accumbens-area was positively correlated with the MoCA scores (*P* < 0.05); the nucleus volume in left and right thalamus-proper, left and right caudate, left and right pallidum, and right accumbens-area was negatively correlated with the frailty index scores (*P* < 0.05). MoCA, Montreal Cognitive Assessment; FI, Frailty index.

After adjusting for age, sex, and education, the volumes of the left and right thalamus proper, right pallidum, and right accumbens area were positively correlated with the MoCA scores (*P* < 0.05); the volumes of the left and right thalamus proper, left and right caudate, left and right pallidum, and right accumbens area were negatively correlated with the frailty index scores (*P* < 0.05; Figure 4, Table 4).

**Table 4:** Correlation of between the volumes of subcortical nuclei and MoCA, frailty index scores.

	MoCA		Frailty Index	
	R	P	R	P
Left-Thalamus-proper	0.378*	0.008	−0.487*	<0.001
Left-Caudate	0.279	0.055	−0.464*	0.001
Left-Pallidum	0.227	0.121	−0.371*	0.009
Left-Accumbens-area	0.11	0.458	−0.075	0.613
Right-Thalamus-proper	0.45*	0.001	−0.475*	0.001
Right-Caudate	0.216	0.139	−0.363*	0.011
Right-Pallidum	0.349*	0.015	−0.403*	0.005
Right-Accumbens-area	0.296*	0.041	−0.33*	0.022

*P < 0.05 significant correlation coefficients.

## Discussion

Cognitive frailty is a clinical condition characterized by the simultaneous presence of both physical frailty and cognitive impairment but without concurrent Alzheimer disease or other dementia. However, there may be some extent of association with physical frailty or cognitive impairment through some common mechanisms. However, the underlying mechanisms of CF remain unclear. In the present study, we investigated the brain structural characteristics in the subcortical nuclei between adults with CF and matched healthy older adults by using DTI technology. The findings of this study showed that there was an obvious decrease in the volume of five brain subcortical nuclei, including the bilateral thalami, left caudate, right pallidum, and right accumbens area in older adults with CF compared to HCs. Furthermore, the volumes of more than five subcortical nuclei were negatively correlated with the frailty index, whereas the volumes of the bilateral thalami, right pallidum, and accumbens were positively correlated with MoCA scores. We also found that those with CF showed decreased FA and RA values in the left thalamus compared to the HCs. These findings suggest that structural atrophy or impairment in some subcortical nucleus areas may be a potential mechanism for CF.

Previous studies have confirmed that the frailty index had significant relationships with subcortical and cortical atrophy but not with changes in white matter in older adults with cognitive decline (Del Brutto et al., 2017; Gallucci et al., 2018). As an important subcortical nucleus, the thalamic nucleus adjacent to the third ventricle connects to its cortex, subcortex, and cerebellum. The thalamic nucleus is a key node in the neural network of hippocampal and cortical structures and is a fundamental substrate for human cognitive functions, especially the formation of memory, information processing, and executive function (Minagar et al., 2013; Fama and Sullivan, 2015; Dolleman-van der Well et al., 2019). Abnormal thalamic macrostructure, microstructure, or neural connectivity is widely associated with some clinical manifestations, such as cognitive impairments, motor deficits, and physical frailty (Fama and Sullivan, 2015). Studies of multimodal MRI have demonstrated that changes in the thalamus, including volume shrinkage, microstructural degradation, and integrity and connectivity decline, are associated with cognitive decline in elderly individuals (Hughes et al., 2012). In addition, a study reported that shrinkage of thalamic volume was positively correlated with gait speed and grip strength in frail populations, such as those with human immunodeficiency virus infection (Kallianpur et al., 2016). The present study found that the volumes of the bilateral thalami were highly correlated with cognitive function and the frailty index, especially the right thalamus, in older adults with CF. Our findings were in line with previous studies. Brain microstructure, tissue quality, and fiber integrity can be assessed with DTI by measuring the magnitude and orientation of water diffusion within the image volume unit (Jones et al., 2013). Fractional anisotropy and RA are two of the most commonly used scalar measurement methods of anisotropy in DTI. Published studies have shown that FA has superior noise immunity relative to RA (Hasan et al., 2004). Fractional anisotropy may assess the integrity of white matter fibers by the orientation of water diffusivity (Truong et al., 2014). Moreover, this modality of FA could also be used to assess the microstructure of gray matter because it likely reflects the integrity of the neuronal cell wall and intercellular membranes (Whitwell et al., 2010). ADC mapping can quantitatively evaluate changes in brain water diffusivity and improve the performance of automatic morphological diagnosis. Our results showed that cognitive decline and physical frailty were highly correlated with the FA and RA values of the thalamus after adjustment for age and some other factors, but we did not find significant differences in the ADC mapping of the subcortical nucleus between the two groups. This might be attributed to the different compartmentalization and impact of diffusion time on the ADC of different molecules under limited diffusion conditions (Kan et al., 2012). Nevertheless, it is possible that thalamic microstructural damage and atrophy may be predictors of CF.

The current study also found that the caudate volume in the CF group was lower than that in the healthy control group, and the caudate volume, especially the left caudate, had a negative correlation with physical frailty. The caudate and putamen constitute the neostriatal nucleus that receives projections from the cerebral cortex and sends them to the brain stem and spinal cord, which play a complex role in the function of the motor regulation system (Jane, 2020). A previous study reported that the caudate nucleus importantly contributed to body and limb posture, as well as the accuracy and speed of directed movements (Villablanca, 2010). These findings may explain why volume changes in the caudate nucleus are related to the frailty index and not cognitive impairment in the current study. Another study reported that the caudate nucleus may play a significant role in brain processing of muscle pain (Maeda et al., 2011), which could be related to physical frailty. In addition, the abnormal functional connection between the caudate nucleus and prefrontal lobe was involved in motor inhibition related to obsessive–compulsive disorder, which also showed the importance of the caudate nucleus in motor control (Fineberg et al., 2018).

The globus pallidus is a principal component of the basal ganglia. The globus pallidus contains a rich concentration of neuropeptides and endogenous cannabinoids, which are important neural signaling molecules that affect brain function, including motivation, cognition, and action and are related to nervous system diseases (Chen et al., 2020; Jakimovski et al., 2020). A study in patients with first-episode psychosis showed that worse cognitive performance was associated with decreased gray matter in the right globus pallidus (Dempster et al., 2017). The globus pallidus plays an important role in the pathogenesis of cognitive and behavioral disorders associated with Huntington disease (Singh-Bains et al., 2016a). Moreover, Nombela et al. (2019) reported that patients with Parkinson disease were treated with deep brain stimulation of the globus pallidus, and improvements in motor function were observed at the 2- to 3-month follow-up. The present study found that the pallidal volume in older adults with CF was lower than that in HCs and was correlated with cognitive decline and physical frailty. These findings were consistent with previous studies, indicating that the globus pallidus plays an important role in regulating cognition and movement. In this study, we also found that volume of the right accumbens area in older adults with CF was smaller than that in HCs and was related to both physical frailty and cognitive impairment, which might indicate that the accumbens area may participate in the regulation of cognitive and physical frailty together with the thalamus, caudate, and pallidum. According to Singh-Bains et al. (2016b), the globus pallidus and nucleus accumbens contribute to “limbic-motor integration” together by transforming the marginal motor signal into motor output. Therefore, the volume atrophy of the globus pallidus and accumbens could also be related to this. In fact, the human nucleus accumbens is an important structure in maintaining normal cognition, motivation, and emotional processes and is involved in some neuropsychiatric disorders (Cartmell et al., 2019). There was also a negative correlation between cognitive function and accumbens volume (Mavridis et al., 2011).

## Limitations

Our study had several limitations. First, the sample size in this study was relatively small. Although we implemented methodological approaches to minimize small sample effects by matching patients and controls in age, sex, and education, it was possible not to detect group differences in certain tracts because of the lack of statistical power. In the future, a study with a larger sample size is needed to replicate our findings. Second, this study was a cross-sectional design, which limits causal and temporal inferences. Longitudinal follow-up studies will be required to better assess the relationship of neuroimaging changes and CF. Third, this study focused only on the main subcortical gray matter nuclei and excluded the cortex and white matter, but in fact, the realization of brain function is a reflection of the overall structure. Fourth, the volume of those subcortical nuclei was inevitably influenced by their part surrounding structure, because of lack of partial volume correction in image data processing. Even so, our current findings provide new insights into the pathological progression of CF.

## Conclusion

The present study shows that microstructural atrophy of the subcortical nuclei occurs in the brains of older adults with CF compared to those of healthy subjects, and the atrophy was correlated with the degree of cognitive decline and physical frailty. These findings demonstrate the involvement of subcortical nuclei in the pathological progression of CF.

## Data Availability Statement

The datasets used and/or analyzed during the current study are available from the corresponding author upon request.

## Ethics Statement

The studies involving human participants were reviewed and approved by the second people’s hospital affiliated to Fujian University of Traditional Chinese Medicine. The patients/participants provided their written informed consent to participate in this study.

## Author Contributions

GZ and LC conceived and designed the study. JT was in charge of coordination and direct implementation. MW and RX wrote the manuscript. MW, RX, HL, PQ, JH and YY managed the recruitment and the follow-up. All authors read and approved the final manuscript.

## Conflict of Interest

The authors declare that the research was conducted in the absence of any commercial or financial relationships that could be construed as a potential conflict of interest.

## Footnotes

^1^https://www.softpedia.com/get/Science-CAD/MRIConvert.shtml

^2^http://www.freesurfer.net

^3^https://fsl.fmrib.ox.ac.uk/fsl/fslwiki/
